# Awareness and knowledge of cervical cancer and its prevention among the nursing staff of a tertiary health institute in Ahmedabad, Gujarat, India

**DOI:** 10.3332/ecancer.2012.270

**Published:** 2012-09-25

**Authors:** V Shah, S Vyas, A Singh, M Shrivastava

**Affiliations:** 1 Department of Community Medicine, GCS Medical College, Ahmedabad, Gujarat, India; 2 Department of Community Medicine, AMC MET Medical College, Ahmedabad, Gujarat, India; 3 Smt NHL Municipal Medical College, Ahmedabad, Gujarat, India

**Keywords:** *cervical cancer*, *knowledge*, *nursing staff*, *PAP test*

## Abstract

**Background:**

Carcinoma of the cervix is the second most common cancer in women worldwide, while it is the commonest cancer among Indian women. Awareness regarding cervical cancer and its prevention is quite low amongst Indian women. The Pap test is a simple and cost effective technique for early diagnosis of cervical cancer. It is necessary to make nursing staff aware of cervical cancer, so that they can impart knowledge regarding cervical cancer and its prevention to the general public.

**Aims and objectives:**

(1) To assess the knowledge level regarding symptoms, risk factors, prevention and screening of cervical carcinoma among nursing staff. (2) To find out the behaviour of respondents regarding prevention and screening of cervical carcinoma.

**Materials and methods:**

A cross-sectional interview-based survey regarding knowledge levels about cervical carcinoma was conducted among the nursing staff from one of the tertiary health institutes of Ahmedabad, India. A structured questionnaire with multiple choices was used for data collection. Provision for open-ended responses was also made in the questionnaire. Department-wise stratification was carried out, and thereafter 15% of the total nursing staff from all departments were selected randomly so as to include a total of 100 nurses in the current study. Data entry was done in Microsoft Excel. SPSS statistical software was used to generate statistical parameters like proportion, mean, standard deviation, etc. The *Z* test was used as a test of significance, and a *P* value of <0.05 was considered as the level of significance.

## Introduction

According to the International Agency for Research on Cancer (IARC), India has the highest number of cervical cancer cases in the world. There are an estimated 1,32,000 new cases and 74,000 deaths each year which occur due to cervical cancer in India [1]. Sexually transmitted infection with human papilloma virus (HPV) is fundamental to the development of carcinoma of the cervix. HPV prevalence increases with multiple sexual partners and poor genital hygiene. Of the 100 HPV types, 18 have been categorized as high-risk types for cervical cancer, while the rest are low-risk types [2]. Cervarix® made by Glaxo SmithKline (GSK) is a bivalent vaccine that protects against HPV strains 16 and 18, and Gardasil® by Merck is a quadrivalent vaccine that protects the individual against HPV strains 16, 18, 6 and 11. These two types of vaccine are available for use by the community with private health care providers. However, there is no provision for HPV vaccine at the institute where the study has been carried out. HPV types 16 and 18 are said to account for approximately 70% of all cervical cancer cases in India [3]. The Programme for Appropriate Technology in Health (PATH), a USA-based not for profit non-governmental organization (NGO), has been undertaking post-licensing observational studies on HPV vaccines in India on coverage, acceptability, feasibility and costs of the vaccines in two Indian states, Gujarat and Andhra Pradesh, funded by the Bill & Melinda Gates Foundation [4]. The study was suspended in April 2010 by the Government of India amid public concerns about safety. Currently, PATH and the Indian government are investigating whether to implement a HPV vaccination program [5].

Cervical cancer is a deadly disease once it reaches the invasive stages, but out of all the female genital tract cancers, it is the only preventable cancer if detected at its early stages. Population-based screening with Pap smear is an important secondary preventive measure for cervical cancer that leads to a high-cure rate among cervical cancer patients. The facilities to carry out Pap smear are available in the institute where the study has been carried out. Also, under the National Cancer Control Programme, screening camps for early detection of cervical cancer are organized in various regions of Gujarat at regular intervals by the Gujarat Cancer Research Institute which is one of the regional cancer care institutes of India.

A recent qualitative study [6] reported a low level of knowledge on HPV and cervical cancer among children, parents, teachers, community leaders and even health service providers of four developing countries (India, Peru, Uganda and Vietnam). Very similar results, i.e. lack of proper knowledge regarding cervical cancer, were found in several studies conducted in other countries in the world [7–11].

Nurses can provide health promotion counselling to the patients they serve in their day-to-day practice. They can fulfil a key role in health promotion and disease prevention, and they are in an ideal position to provide health education to young girls and women. It is necessary to make the nursing staff aware about cervical cancer, who can impart knowledge regarding cervical cancer and its prevention to the general public. The present study was carried out among the nursing staff of a tertiary health institute in order to assess their knowledge regarding cervical cancer.

## Materials and methods

A cross-sectional study was carried out among the nursing staff of a tertiary health institute in Ahmedabad, India. The duration for the study was from March to June, 2006. A total of 620 nursing staff were enrolled under the institute at the time of study. 15% of staff were randomly selected using a table of random numbers after department-wise stratification. The calculated sample size was 93 but practically a total of 100 nurses were selected for the study. Verbal-informed consent was sought from the study subjects. A 15-item structured questionnaire was designed. However, provision for inclusion of open-ended responses was also made in the format. The selected nurses were interviewed by the investigator for seeking information about the socio-demographic profile of the respondents, their knowledge about symptoms, risk factors and prevention, their attitude and utilization of Pap smear as a screening device for carcinoma cervix. Pre-testing of the questionnaire was done on 10 respondents; after which necessary changes were made, and the questionnaire was re-administered. Data entry was done, and SPSS statistical software was used to generate statistical parameters like proportion, mean, standard deviation, etc. *Z* test was used as a test of significance, and *P* value of <0.05 was considered as level of significance.

## Result

Out of 100 staff nurses, 52% belonged to the age group of 41–50 years. The mean age of the study population was 46 years. The majority of respondents (89%) were married (Table 1). 69% of respondents had some knowledge of cervical carcinoma. As per information regarding knowledge of the symptoms of cervical cancer, 65 (94.2%) respondents stated vaginal discharge as one of the symptoms. The percentages of respondents who mentioned menstrual abnormality and pain as symptoms were 86.9 and 66.6, respectively. Only eight (11.5%) respondents were aware of multiple sexual partners as one of the risk factors of cervical carcinoma. Out of 69 respondents who had some knowledge regarding cervical carcinoma, 61 (88.4%) had knowledge regarding Pap test as one of the preventive measures (Table 2). Out of 61 staff nurses who knew about Pap test, only five (8%) had undergone Pap test (Table 3).

## Discussion

The present study was conducted among staff nurses in order to evaluate their knowledge regarding cervical carcinoma. 69% of staff nurses had some knowledge related to cancer of the cervix. In the present study, 86.9 and 94.2% mentioned menstrual abnormality and abnormal vaginal discharge, respectively, as symptoms of cervical cancer, while in a study by Nganwai *et al* [7], 77.7 and 92.4% knew that common symptoms of cervical cancer include post-coital bleeding, inter-menstrual bleeding and abnormal leucorrhoea or blood-stained vaginal discharge. A similar finding (menstrual abnormality -80.6%) was found in a study by Anya *et al* [8] among female health personnel.

In our study, only 11.5% mentioned multiple sexual partners as one of the risk factors, while in a study of Ali *et al* [9], 45% mentioned multiple partners and other promiscuous behaviour as the most common risk factor. In a study carried out by McCarey *et al* [10], 41% of nurses mentioned multiple sexual partners as a risk factor for cervical cancer. In the present study, 73.9% mentioned early age at pregnancy as one of the risk factors for cervical cancer. Nganwai *et al* [7] in their study mentioned that 81.8 and 85.6% of respondents knew that first sexual intercourse at a young age and having multiple sexual partners were risk factors for cervical cancer.

In the present study, knowledge regarding Pap test was present in 88.4% of respondents. Similar findings (83%) were documented in a study carried out by Mutyaba *et al* [11]. In a study by Ali *et al* [9], 75% knew that Pap smear is the screening test for cervical cancer.

In the present study, only 5 (5%) respondents underwent Pap test. The same result (5.5%) was there in a study by Udigwe [12]. In a study carried out by Nganwai *et al* [7], 56.4% underwent Pap smears every year.

## Conclusions

Our data suggest that levels of knowledge and understanding of cervical cancer as well as its preventable nature should be improved. Continuing nurse education may contribute to strengthen cervical cancer screening programs. Nursing staff, if properly aware of this disease, can educate the masses and hence increase health-seeking behaviour in women.

## Figures and Tables

**Table 1:** Socio-demographic profile of study population

Socio-demographic variable	Frequency	Percentage
Age		
21–30	6	6
31–40	14	14
41–50	52	52
51-60	28	28
Marital status		
Married	89	89
Unmarried	9	9
Widow	2	2
Socio-economic class ^a^		
Class 1	85	85
Class 2	15	15
Total	100	100

a Socio-economic class as per the modified Prasad classification.

**Table 2:** Knowledge regarding various aspects of carcinoma cervix

Knowledge about carcinoma cervix	Frequency	Percentage	*Z* value	*P* value
Symptoms				
Menstrual abnormality	60	86.5	1.4	>0.05
Vaginal discharge	65	94.2	1	–
Pain	46	66.6	4.3	<0.05
Others^a^	28	40.5	8.6	<0.05
Risk factors				
Early marriage	49	71.1	0.3	>0.05
Early pregnancy	51	73.9	1	–
Repeated pregnancy	43	62.3	1.4	>0.05
Oral contraceptives	12	17.3	6.9	<0.05
Multiple sexual partners	8	11.5	6.5	<0.05
Preventive measures				
Good genital hygiene	57	82.6	5.9	<0.05
Use of condom	59	85.5	5.7	<0.05
PAP test	61	88.4	1	–
Total	69	100		

a Others included post-coital bleeding, bladder and rectal involvement, weight loss and loss of appetite

**Table 3:** Knowledge regarding various aspects of carcinoma cervix

Knowledge about PAP test as screening test for carcinoma cervix	Underwent PAP test		Total
	Yes, *n* (%)	No, *n* (%)	*n* (%)
Present	5 (8)	56 (92)	61 (100)
Absent	0 (0)	39 (100)	39 (100)
Total	5 (5)	95 (95)	100 (100)

Note: *Figure* in parenthesis include row-wise percentages
